# EOP, a Newly Synthesized Ethyl Pyruvate Derivative, Attenuates the Production of Inflammatory Mediators via p38, ERK and NF-κB Pathways in Lipopolysaccharide-Activated BV-2 Microglial Cells

**DOI:** 10.3390/molecules191219361

**Published:** 2014-11-25

**Authors:** Soon Min, Sandeep Vasant More, Ju-Young Park, Sae-Bom Jeon, Shin Young Park, Eun-Jung Park, Sung-Hwa Yoon, Dong-Kug Choi

**Affiliations:** 1 Department of Biotechnology, College of Biomedical and Health Science, Konkuk University, Chungju 380-701, Korea; 2 Department of Molecular Science and Technology, Ajou University, Suwon 443-749, Korea; 3 Branch of Immune and Cell Therapy, National Cancer Center, Goyang 410-769, Korea

**Keywords:** ethyl pyruvate derivative, lipopolysaccharide, microglia, neuroinflammation, neurodegenerative diseases, primary microglia

## Abstract

Microglia-induced neuroinflammation is an important pathological mechanism influencing various neurodegenerative disorders. Excess activation of microglia produces a myriad of proinflammatory mediators that decimate neurons. Hence, therapeutic strategies aimed to suppress the activation of microglia might lead to advancements in the treatment of neurodegenerative diseases. In this study, we synthesized a novel ethyl pyruvate derivative, named EOP (*S*-ethyl 2-oxopropanethioate) and studied its effects on lipopolysaccharide (LPS)-induced production of nitric oxide (NO) in rat primary microglia and mouse BV-2 microglia. EOP significantly decreased the production of NO, inducible nitric oxide synthase, cyclooxygenase and other proinflammatory cytokines, such as interleukin (IL)-6, IL-1β and tumor necrosis factor-α, in LPS-stimulated BV-2 microglia. The phosphorylation levels of extracellular regulated kinase, p38 mitogen-activated protein kinase, and nuclear translocation of NF-κB were also inhibited by EOP in LPS-activated BV-2 microglial cells. Overall, our observations indicate that EOP might be a promising therapeutic agent to diminish the development of neurodegenerative diseases associated with microglia activation.

## 1. Introduction

The central nervous system (CNS) has been assumed to be an immunologically-restricted organ because the blood-brain barrier eliminates most of the components of the immune system and thereby limits its inflammatory capacity and prevents lymphatic infiltration [1]. Growing evidence suggests that neuroinflammation is one of the important pillars in the pathogenesis of neurological disorders, such as stroke, Parkinson’s disease, Alzheimer’s disease, multiple sclerosis and Huntington’s disease [1]. Microglia are resident neuroprotective immune cells in the brain that support neural growth and metabolism and are scavenging agents that are toxic to neurons [2]. Excess activation of microglia due to brain injury or exposure to chemical stimuli causes excessive release of various neurotoxic substances, such as nitric oxide (NO), prostaglandin E2, superoxide and proinflammatory cytokines, such as interleukin-6 (IL-6), IL-1β and tumor necrosis factor-α (TNF-α) [3], which have been implicated in various neurodegenerative diseases.

Ethyl pyruvate (EP) is a simple aliphatic ester derived from the endogenous metabolite, pyruvate [4]. EP is more stable and safer than pyruvate for inhibiting the generation of reactive oxygen species (ROS) and inflammation [5]. EP mitigates damage caused by hemorrhagic shock, sepsis, acute pancreatitis, burn injury, radiation injury, coagulation and ischemia [6,7,8,9,10,11,12]. EP has also been reported to benefit the angiogenesis of tumors by inhibiting NF-κB signaling and directly targeting the p65 subunit of the transcription factor [5,13]. EP also attenuates secretory, adhesive and signaling events indicative of the early inflammatory response in endothelial cells [14]. In pathologies related to the CNS, EP attenuates kainic acid-induced neuronal cell death in the mouse hippocampus [15], confers potent neuroprotection against neonatal H-I brain injury via its anti-cell death and anti-inflammatory actions [16], protects PC12 cells against dopamine by modulating key signaling pathways of apoptosis and inhibiting intercellular oxidative stress [17] and protects nigrostriatal dopaminergic neurons by modulating activation of glia in a mouse model of Parkinson’s disease [18].

Murine BV-2 microglia have been used to simulate primary microglia in research related to neuroinflammatory disorders [19]. Thus, determining a way to suppress lipopolysaccharide (LPS)-induced inflammation in BV-2 microglia could be a therapeutic strategy to treat neuroinflammatory diseases [20,21]. We synthesized novel EP derivatives with the aim of producing an anti-inflammatory profile. Among the eight novel synthetic EP derivatives, we observed that S-ethyl 2-oxopropanethioate (EOP) significantly suppressed the inflammatory response in LPS-activated BV-2 cells. Pre-treating BV-2 microglial cells with EOP decreased LPS-induced generation of NO and cyclooxygenase-2 (COX-2) along with inflammatory cytokines, such as IL-6, IL-1β and TNF-α. A subsequent examination of molecular pathways revealed that the anti-neuroinflammatory activity of EOP occurred by inhibiting phosphorylation of p38 and extracellular regulated kinase-mitogen activated protein kinase (ERK-MAPK) and by suppressing nuclear levels of the nuclear factor (NF)-κB p65 subunit in LPS-stimulated BV-2 cells. These results widen our understanding of the anti-neuroinflammatory effects of EOP and suggest a possible new pharmacological agent for treating neuroinflammatory diseases.

## 2. Results and Discussion

### 2.1. Synthesis of the Novel Ethyl Pyruvate Derivative, EOP

**Figure 1:**
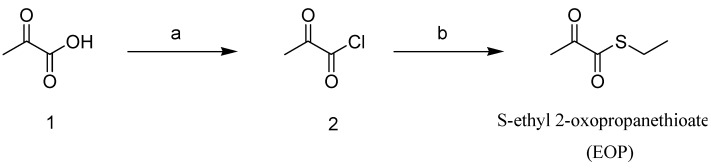
Synthesis of the novel S-ethyl 2-oxopropanethioate, ethyl pyruvate derivative, EOP.

We synthesized eight novel clovamide derivatives, and EOP was the most efficacious among them. As shown in Figure 1, EOP was synthesized from commercially available pyruvic acid 1. After pyruvic acid 1 was converted to 2-oxopropanoyl chloride 2 using α,α-dichloromethyl methyl ether, the resulting 2-oxopropanoyl chloride 2 was reacted with ethanethiol to produce the crude product, which was purified by silica gel chromatography with dichloromethane to give EOP.

Pyruvate is an anionic form of a simple alpha-keto acid that plays a key role in metabolism as a product of glycolysis and is the starting substrate for the tricarboxylic acid cycle [22]. Although pyruvate is a crucial intermediate metabolite, it is also an important endogenous scavenger of ROS, particularly hydrogen peroxide (H_2_O_2_) [23]. Pyruvate is unstable in aqueous solvent, and it spontaneously undergoes cyclization and condensation reactions to form different products, some of which may be toxic, thus limiting its therapeutic potential [24]. EP is an ester formed by condensation of pyruvic acid with ethanol [22], which is not only stable in aqueous solution, but also shows antioxidant effects [25]. Thus, many reports have shown that EP has anti-inflammatory, anti-apoptotic and antioxidant effects [22]. In this study, we synthesized eight novel synthetic EP derivatives and tested their anti-neuroinflammatory properties in LPS-stimulated rat-derived primary microglial cells and murine BV-2 microglial cells.

### 2.2. Effect of EOP on LPS-Induced Production of NO and Cytotoxicity in Rat Primary and Murine BV-2 Microglia

**Figure 2:**
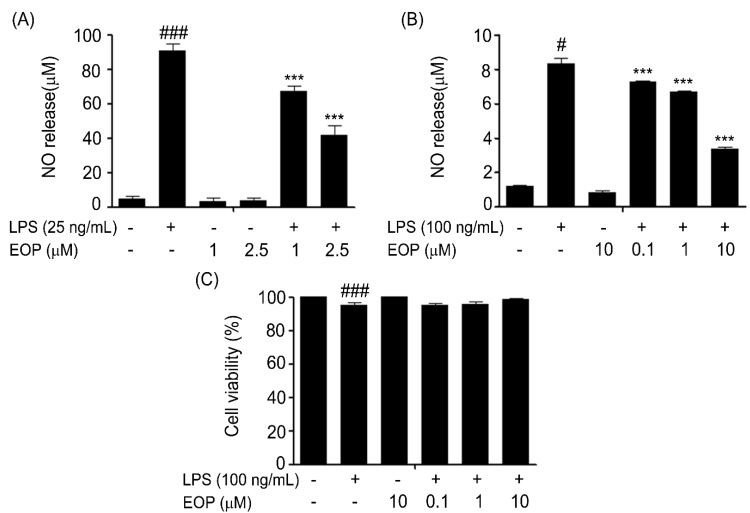
The effect of EOP on cell viability and production of nitric oxide (NO) in lipopolysaccharide (LPS)-stimulated mouse BV-2 microglial cells. (**A**) Rat primary microglia and (**B**) BV-2 cells were pre-treated with different concentrations of EOP (1 and 2.5 μM) and (0.1, 1 and 10 μM) for 60 min before incubating with LPS (25/100 ng/mL) for 24 h. Nitrite content in the culture media was measured using the Griess reaction, and the results were expressed as a percentage of released NO from LPS-stimulated BV-2 cells. (**C**) Cells were treated with various doses of EOP (0.1, 1 and 10 μM) for 60 min before incubating with LPS (100 ng/mL) for 24 h. The cytotoxicity of EOP was assessed by the MTT assay, and the results are expressed as a percentage of surviving cells over control cells. Data are expressed as a percentage of the control. Data are the mean ± standard error (SEM) of three independent experiments. ^###^ *p* < 0.001 compared with the control group and *** *p* < 0.001 compared with the LPS-treated group. Significance was determined by one-way analysis of variance followed by Bonferroni’s multiple comparison test.

To evaluate the effect of EOP on LPS-induced NO production, primary microglial cells were treated for 1 h with two doses of EOP (1 and 2.5 µM) and then with or without LPS 25 ng/mL for 24 h. The nitrite released from the cells into the medium was measured by the Griess reaction. As shown in Figure 2A, stimulating rat primary microglial cells with 25 ng/mL LPS for 24 h caused significant release of NO compared with that by the control. Pretreatment with 1 and 2.5 µM EOP decreased the LPS-induced increase in NO production. We also demonstrated the effect of EOP on LPS-induced release of NO in BV-2 microglial cells. Murine BV-2 microglial cells were treated for 1 h with various doses of EOP (0.1, 1 and 10 µM) and exposed to LPS (100 ng/mL) for 24 h. As demonstrated in Figure 2B, exposure to LPS significantly stimulated the release of NO compared to that in the control group. Cells treated *perse* with EOP did not show elevated levels of NO. Pre-treatment of BV-2 microglia with EOP at 0.1, 1 and 10 µM significantly reduced NO levels.

The MTT assay was performed to explore any cytotoxic effects of EOP on BV-2 microglia cells. BV-2 cells were pre-treated with LPS (100 ng/mL) with or without various doses of EOP (0.1, 1 and 10 µM) for 24 h. LPS (100 ng/mL) in combination with EOP did not reduce the viability of microglial cells (Figure 2C). Hence, our results indicate that EOP had a significant inhibitory effect on LPS-stimulated NO release in rat primary cells and murine BV-2 microglial cells.

Microglial cells constituting the brain’s immune system are indispensable for assuring neuroprotection in a normal and pathological brain [26]. In anticipation of injury to neurons, as evident in neurodegenerative disorders [27] and experimental brain injury [28], microglia produce a multitude of proinflammatory cytokines and chemokines [29]. If this inflammatory reaction is prolonged, it causes severe damage to neuron health and their functions. Hence, timely management of brain inflammation is one of the therapeutic strategies to combat neurodegenerative diseases. As NO is a crucial proinflammatory mediator that plays an important role in neuroinflammatory diseases, we tested the effect of EOP on NO release from rat primary microglia and BV-2 cells exposed to LPS [30]. We discovered that EOP decreased LPS-stimulated release of NO in rat primary microglia and BV-2 cells in a dose-dependent manner.

### 2.3. EOP Attenuates LPS-Mediated Inducible Nitric Oxide Synthase (iNOS) and COX-2 Expression in BV-2 Microglia

**Figure 3:**
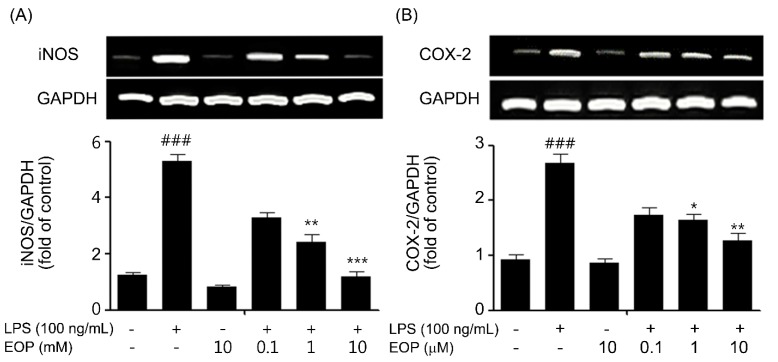

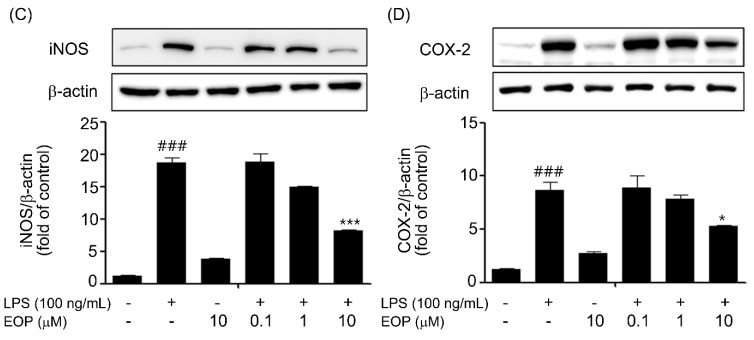
EOP inhibits lipopolysaccharide (LPS)-stimulated release of inducible nitric oxide synthase (iNOS) and cyclooxygenase-2 (COX-2) in BV-2 microglial cells. BV-2 microglial cells were pre-treated with the indicated concentrations of EOP for 60 min before incubating with LPS (100 ng/mL) for 6 h. Quantified data are shown in the lower panel. (A) iNOS and (B) COX-2 mRNA levels were normalized to those of GAPDH and expressed as the relative change in comparison to the LPS treatment. BV-2 cells were pretreated with EOP (0.1, 1 and 10 μM) for 60 min and then stimulated with LPS (100 ng/mL) for 18 h. Quantification of (C) iNOS and (D) COX-2 was performed by normalization with β-actin and expressed as the relative change in comparison to the LPS treatment. Data are the mean ± standard error (SEM) of three independent experiments. ^###^ *p* < 0.001 compared with the control group; *** *p* < 0.001, ** *p* < 0.01 and * *p* < 0.05 compared with the LPS-treatedgroup. Significance was determined by one-way analysis of variance followed by Bonferroni’s multiple comparison test.

Activated microglial cells kill neurons through NO derived from iNOS by inhibiting neuronal respiration [5]. Furthermore, iNOS and COX-2 are key enzymes released in LPS-stimulated microglial cells and are observed in various neurodegenerative diseases [31]. Therefore, we explored the effects of EOP on LPS-induced expression of iNOS and COX-2 in BV-2 microglial cells. PCR and immunoblotting studies were conducted to detect iNOS and COX-2 mRNA and protein levels. BV-2 cells were pretreated with EOP (0.1, 1 and 10 µM) for 1 h and then exposed to LPS (100 ng/mL) for another 6 and 24 h for PCR and immunoblotting studies, respectively. As shown in Figure 3, pre-treatment with EOP at various doses significantly reduced LPS-induced iNOS mRNA (Figure 3A) and protein expression (Figure 3C) in a dose-dependent fashion. Similarly, we found that LPS increased COX-2 mRNA (Figure 3B) and protein levels (Figure 3D). Treatment with different doses of EOP dose-dependently inhibited LPS-induced mRNA and protein levels of iNOS and COX-2 in BV-2 microglial cells. Our results agree with earlier reports showing that EP suppressed NO and iNOS [5,32].

### 2.4. EOP Mitigates LPS-Induced Expression of Pro-Inflammatory Cytokines in Microglial Cells

Over-production of NO has been correlated with cell death, and NO is reported to be regulated by NF-κB, which performs a key role mediating the early stages of immune and inflammatory responses [33]. In addition, NF-κB is also documented to perform the generation of pro-inflammatory cytokines, such as, tumor necrosis factor-α (TNF-α), interleukin-1β (IL-1β) and interleukin-6 (IL-6) [34], which ultimately result in exacerbating neurotoxicity [35]. 

**Figure 4:**
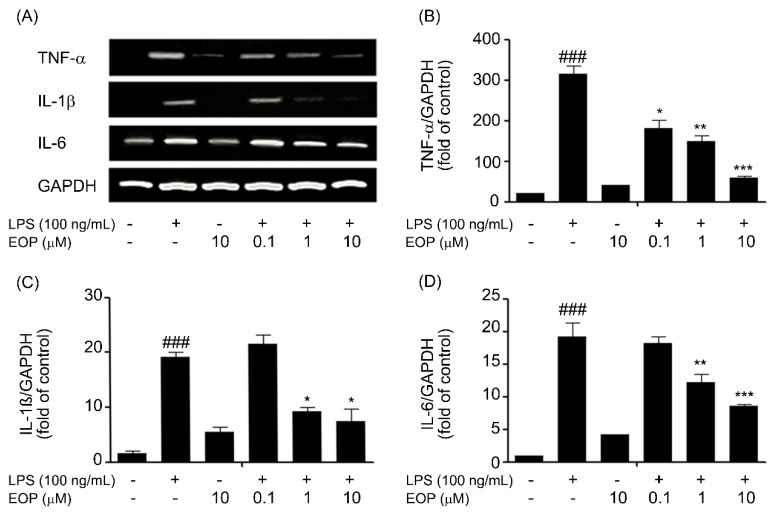
EOP attenuates pro-inflammatory cytokine gene expression in lipopolysaccharide (LPS)-simulated BV-2 microglial cells. BV-2 microglial cells were pre-treated with EOP (0.1, 1 and 10 μM) and then stimulated with LPS (100 ng/mL), and total RNA was isolated at 6 h after the treatment. (**A**) The mRNA levels of TNF-α, IL-1β and IL-6 were determined by reverse transcription polymerase chain reaction and subjected to densitometric quantification. Levels of (**B**) TNF-α, (**C**) IL-1β and (**D**) IL-6 mRNA were normalized to GAPDH levels and expressed as the relative change in comparison to the LPS treatment. ^###^ *p* < 0.001 compared with the control group; *** *p* < 0.001, ** *p* < 0.01 and * *p* < 0.05 compared with the LPS-treatedgroup. Significance was determined by one-way analysis of variance followed by Bonferroni’s multiple comparison test.

Hence, based on earlier reports that proinflammatory cytokines released from microglia are necessary for neuronal cell death [36], we examined the effects of EOP on suppressing the pro-inflammatory cytokines, TNF-α, IL-6 and IL-1β, in BV-2 microglia. BV-2 microglia cells were pre-treated with EOP (0.1, 1 and 10 μM) for 60 min with or without exposure to LPS (100 ng/mL) for 6 h. The results indicated that BV-2 microglial cells exposed to LPS for 6 h caused a significant increase in the expression of TNF-α, IL-1β and IL-6 compared to that in the vehicle group (Figure 4). Pre-treatment with EOP at various doses decreased LPS-induced TNF-α, IL-1β and IL-6 mRNA levels in a dose-dependent fashion (Figure 4B–D). Our results suggest that EOP suppressed production of TNF-α, IL-6 and IL-1β at the transcriptional level in response to LPS exposure to BV-2 microglial cells. Our results were in parallel with a report that EP inhibits cytokines [37].

### 2.5. EOP Inhibits LPS-Induced Phosphorylation of p38 and ERK MAPK in BV-2 Microglial Cells

**Figure 5:**
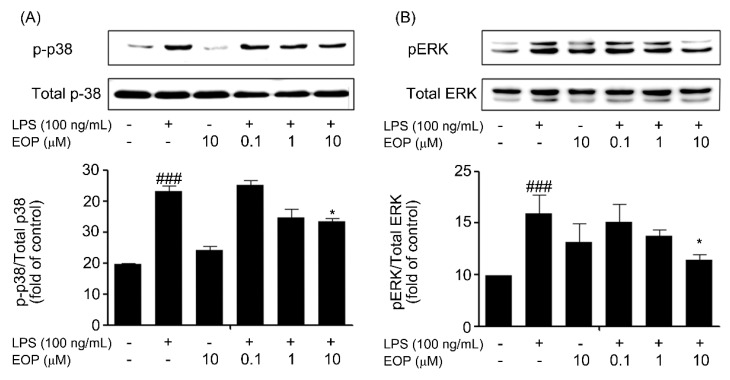
EOP suppresses lipopolysaccharide (LPS)-stimulated p38 and extracellular regulated kinase (ERK) phosphorylation by EOP in BV-2 microglial cells. BV-2 microglial cell lysates were obtained 30 min post-activation with LPS (100 ng/mL) in the absence or presence of EOP (0.1, 1 and 10 μM). (**A**) Levels of phospho-p38 and (**B**) ERK are expressed as a relative change in comparison to the LPS treatment. Densitometric analysis of the p38 and ERK-mitogenactivated protein kinase (MAPK) band are shown in the lower panel. Data are the mean ± standard error of three independent experiments. ^###^ *p* < 0.001 compared with the control group; * *p* < 0.05 compared with the LPS-treated group. Significance was determined by one-way analysis of variance followed by Bonferroni’s multiple comparison test.

MAPK signaling pathways control the production of inflammatory factors in microglial cells [30]. Therefore, we investigated whether EOP influenced these signaling mechanisms in BV-2 microglia cells. BV-2 cells were pre-treated with different doses of EOP (0.1, 1 and 10 µM) for 1 h and then treated with LPS (100 ng/mL) for 30 min. LPS treatment significantly increased p38 and ERK-MAPK phosphorylation at 0.5 h with respect to the vehicle group. EOP inhibited this LPS-stimulated phosphorylation in a dose-dependent fashion (Figure 5A,B), indicating that EOP suppresses p38 and ERK-MAPK signaling in BV-2 microglial cells, which might lead to a decrease in the release of inflammatory mediators. In addition, we did not observe any effect of EOP on LPS-induced expression of the JNK-MAPK signaling pathway. MAPKs are the upstream signaling molecules in inflammatory pathways that integrate and process the cellular responses to a number of diverse extracellular signals, leading to an inflammatory reaction [32]. In our experiments, we observed that EOP significantly decreased LPS-stimulated phosphorylation of p38 and ERK, whereas it had no effect on JNK/MAPK activity. In a comparable report by Jeong *et al.*, EP was also observed to restore ERK phosphorylation in SH-SY5Y cells [28].

### 2.6. Inhibitory Effect of EOP on Phosphorylation of IκB-α and Subsequent Activation and Localization of NF-kB in BV-2 Microglial Cells

**Figure 6:**
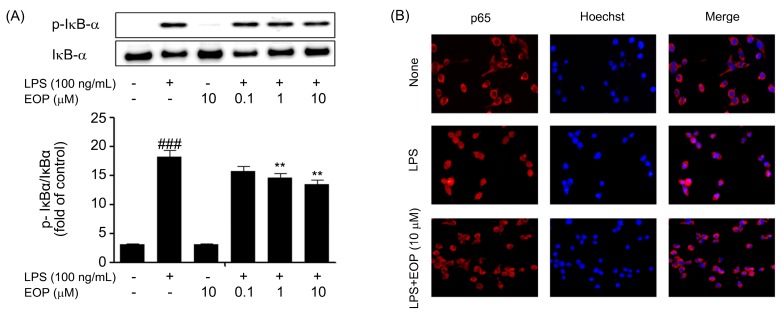
EOP inhibits the degradation of p-IκB-α and further inhibits nuclear translocation of p65-NF-κB in lipopolysaccharide (LPS)-stimulated BV-2 microglial cells. Cells were treated with the indicated doses of EOP, 60 min before LPS (100 ng/mL) treatment. Protein lysates were electrophoresed followed by immunoblotting using (**A**) anti-p-IκB-α. Densitometric analysis of p-IκB-α is shown in the lower panel. Results are expressed as a ratio of p-IκB-α/T-IκB-α. BV-2 microglial cells were seeded at a density of 5 × 10^4^ cells/well on a 24-well plate. (**B**) Cells were treated with EOP (10 μM) for 60 min and then stimulated with LPS (100 ng/mL) for 30 min. Sub-cellular location of the p65NF-κB subunit was determined by an immunofluorescence assay. Data are the mean ± standard error of three independent experiments. ^###^ *p* < 0.001 compared with the control group; ** *p* < 0.01 compared with the LPS-treated group. Significance was determined by one-way analysis of variance followed by Bonferroni’s multiple comparison test.

In addition to MAPKs, we also examined phosphorylation of IκB-α, which assists in nuclear localization of NF-κB, which further executes important roles in the LPS-stimulated production of pro-inflammatory mediators [38]. Activation of NF-κB consists of pre-phosphorylation of IκB-α followed by nuclear localization of the p65 subunit of NF-κB. Based on these results, we pre-treated microglial cells with or without EOP at different doses for 60 min, followed by exposure to LPS (100 ng/mL) for 30 min. As shown in Figure 6A, activation of BV-2 cells by LPS significantly induced phosphorylation of IκB-α. Levels of the p65 subunit of NF-κB were assessed by an immunofluorescence assay by first treating BV-2 microglial cells with EOP (10 μM) followed by 30 min of LPS exposure. Nuclear localization of the p65 subunit of NF-κB was suppressed by 10 μM EOP (Figure 6C).

We demonstrated that EOP decreased IκB-α phosphorylation, which was followed by localization of the p65 subunit of NF-κB into the nucleus, as confirmed by the immunofluorescence assay. Our data represent the same general trend as shown in earlier experiments, wherein EP suppressed the release of cytokines by inhibiting the NF-κB pathway [16]. In comparison to EOP, EP did not have any effect on IκB-α, which suggests that our compound specifically inhibits degradation of IκB-α to prevent nuclear translocation of NF-κB [13].

## 3. Experimental Section

### 3.1. Reagents

LPS (Escherichia coli 0111:B4), 3-(4,5-dimethylthiazol-2-yl)-2,5-diphenyltetrazolium bromide (MTT), *N*-(1-naphthyl) ethylenediamine dihydrochloride, sulfanilamide, dimethyl sulfoxide, bovine serum albumin, Tween-20 and sodium nitrite were procured from Sigma (St. Louis, MO, USA). Phosphate buffered saline and Dulbecco’s modified Eagle medium (DMEM) were purchased from Gibco/Invitrogen (Carlsbad, CA, USA). Protease and phosphatase inhibitors were purchased from Roche (Indianapolis, IN, USA). Fetal bovine serum (FBS) was supplied by PAA Laboratories Inc. (Etobicoke, ONT, Canada). Antibodies to iNOS, p38, phospho-p38, ERK1/2, phospho-ERK1/2, phospho-IκB-α, IκB-α and β-actin were purchased from Cell Signaling Technology (Danvers, MA, USA). Antibodies for the p65 subunit of NF-κB, nucleolin and COX-2 were obtained from Santa Cruz Biotechnology (Santa Cruz, CA, USA).

### 3.2. Microglial Cell Culture

Primary microglial cells were cultured from the cerebral cortices or substantia nigra of 1–3 day-old Sprague-Dawley rats. Briefly, tissues were triturated into single cells in MEM containing 10% FBS and were plated in 75-cm^2^ T-flasks (0.5 hemisphere/flask) for 2 weeks. The microglia were detached from the flasks by mild shaking and were applied to a nylon mesh to remove astrocytes and cell clumps. Cells were plated in 6-well plates (5 × 10^5^ cells/well) or 60-mm^2^ dishes (8 × 10^5^ cells/dish). One hour later, the cells were washed to remove any unattached cells before they were used for experiments.

The BV-2 microglial cells were originally developed by V. Bocchini and demonstrate both compositional and operational properties of activated microglial cells, as cited in our earlier report [39], and were utilized in this study [40]. The cells were cultured in DMEM consisting of 5% heat-inactivated FBS and a 1% of 50 µg/mL penicillin-streptomycin solution and were maintained in incubator with 5% CO_2_ at 37 °C.

### 3.3. NO and Cell Viability Assay

Primary microglial cells were seeded in 6-well plates (5 × 10^5^ cells/well). The cells were pre-treated with various doses of EOP (0.1, 1 and 10 µM) for 1 h, followed by LPS (25 ng/mL) treatment for 24 h. Similarly, murine BV-2 cells, seeded at 2.5 × 10^4^ cells/mL in 96-well plates, were pre-treated with various EOP concentrations (0.1, 1 and 10 µM) for 1 h followed by LPS (100 ng/mL) treatment for 24 h. The remaining procedures for detecting NO were carried out as described in our previous report [39]. Results are representative of three individual experiments.

BV-2 cell viability was quantified by measuring the formation of formazan crystals. BV-2 cells, seeded at 2.5 × 10^4^ cells/mL in 96-well plates, were pre-treated with various doses of EOP (0.1, 1 and 10 µM) for 1 h followed by LPS (100 ng/mL) treatment for 24 h. The reminder of the procedure was carried out as described in our previous report [41]. Results are representative of three individual experiments.

### 3.4. EOP Synthesis Methodology

2-Oxopropanoyl chloride 2 (2.01 g, 18.9 mmol), which was prepared from pyruvic acid and α,α-dichloromethyl methyl ether according to the procedure reported in the literature [1], was added to a solution of ethanethiol (2.33 g, 37.5 mmol) and triethylamine (5.23 mL, 37.5 mmol) in dichloromethane (40 mL) at 0 °C. 2-oxopropanoyl chloride 2 (2.01 g, 18.9 mmol), which was prepared from pyruvic acid and α,α-dichloromethyl methyl ether according to the procedure reported in the literature [1], was added. The mixture was stirred at room temperature for 4 h and then quenched with 1 N HCl solution. The solution was extracted with ethyl acetate. The organic layer was washed with water and brine, dried over anhydrous sodium sulfate and evaporated in vacuum. The crude residue was purified by silica gel column chromatography to give the title compound as a yellow oil (1.11 g, 45%). ^1^H nuclear magnetic resonance (NMR) (CDCl_3_, δ in ppm): 1.15–1.25 (m, 6H), 2.42 (s, 3H), 3.25–3.30 (q, 2H, *J* = 6.8 Hz), 3.35–3.45 (q, 2H, *J* = 7.2 Hz); 13C NMR (CDCl3, δ in ppm): 12.54, 14.41, 27.52, 39.32, 41.95, 166.07, 198.37; electrospray ionization mass spectroscopy: *m/z* 143.90 ([M+H]^+^).

### 3.5. RNA Isolation and Reverse Transcription Polymerase Chain Reaction (RT-PCR)

RNA isolation was performed using TRIzol in BV-2 cells seeded at 50 × 10^4^ cells/mL and maintained in 6-well plates. For RT-PCR experiments, 2.5 μg RNA was reverse transcribed using a cDNA synthesis kit. cDNA was amplified using targeted primers as mentioned previously [42,43]. As described previously [44], PCR was performed using cDNA as a template for specific targets, and 1% agarose gel electrophoresis was used to analyze the PCR products. The results were representative of three individual experiments.

### 3.6. Western Blot Analysis

BV-2 cells seeded at 40 × 10^4^ cells/mL and cultured in 6-well plates were rinsed twice with ice cold PBS after EOP and LPS treatment. The total cell extract was obtained by adding 80 μL RIPA buffer (1*×* PBS, 1% NP-40, 0.5% sodium deoxycholate and 0.1% SDS, containing fresh protease inhibitor cocktail). In a related experiment, a nuclear extraction kit from Thermo Scientific (Rockford, IL, USA) was used to separate nuclear proteins. Electrophoresis and immunoblotting procedures were carried out as per our previous report [41]. PVDF membranes were incubated overnight with anti-iNOS (1:1000), anti-(inhibitor of κB-α) IκB-α (1:1000), anti-p-IκB-α (1:1000), anti-total-ERK (1:1000), anti-phospho-ERK (1:1000), anti-total-p38 (1:1000), anti-phospho-p38 (1:1000) anti-β-actin (1:2000), anti-p65 subunit of NF-κB (1:500), anti-nucleolin (1:1000) and anti-COX-2 (1:2000) antibodies, followed by a 1-h incubation with horseradish peroxidase-conjugated secondary antibody (1:2000). The LAS-3000 Luminescent Image system (Fuji, Tokyo, Japan) was used to visualize antibody-specific bands on the PVDF membrane. Results are representative of three individual experiments.

### 3.7. Immunocytochemistry

BV-2 microglia cells, seeded at 5 × 10^4^ cells/well on sterile cover slips, were cultured in 24-well plates. The cells were pre-treated with 10 µM EOP for 1 h followed by exposure to LPS (100 ng/mL) for 30 min. Cell fixation on cover slips and further steps were carried out as per our earlier report [41]. Detection of the intracellularly localized p65 sub-unit of NF-κB was performed using a fluorescence microscope.

### 3.8. Statistical Analyses

GraphPad software ver. 5 (Graph Pad Software, La Jolla, CA, USA) was used to carry out statistical analyses. All values are presented as the mean ± standard error. Significant differences between groups were detected by one-way analysis of variance followed by Bonferroni’s multiple comparisons test. *p* < 0.05 was considered statistically significant. ^###^ *p*, and ^#^ *p* is assigned as statistical significance to LPS treated group in comparison to control group. 

## 4. Conclusions

Our results indicate that EOP may act on p38, ERK and NF-κB signaling pathways, which are predominantly, involved in the release of inflammatory mediators in LPS-stimulated BV-2 microglial cells. Therefore, EOP might also prove to be a potential therapeutic candidate for treating inflammation observed in neurodegenerative disorders.
